# Traité Pratique sur les Maladies des Organes Génito-Urinaires

**Published:** 1842-07-01

**Authors:** 


TRAITÉ PRATIQUE SUR LES MALADIES DES ORGANES GÉNITO-URINAIRES. Par le Docteur *Civiale*. Tom. 3, pp. 1438, 8vo. Paris, 1837—42.

A Practical Treatise upon the Diseases of the Genito-urinary Organs. By Dr. *Civiale*. 3 Vols. 8vo.

WE have perused this book with much attention and great satisfaction, and feel desirous of presenting our readers with a faithful analysis of its valuable contents. However severely we may have felt called upon, in our last number to reprobate Dr. Civiale's falsification of facts, in reference to the results of his lithotrity operations, upon the present occasion we observe the development of sound chirurgical principles, based upon able reasoning, and supported by a most extensive experience.

The work does not embrace the venereal diseases and calculous affections of the genito-urinary organs; and is divided into three parts, each occupying a volume. The first relates to stricture and its consequences: the second to the diseases of the neck of the bladder and of the prostate gland: and the third to diseases of the body of the bladder.

An introductory chapter presents us with some general observations upon the anatomy and pathology of the genito-urinary organs. The former need not detain us farther than to observe, that the author represents the urethra, from more than 200 admeasurements, to be much shorter than it is usually understood to be, viz. six inches in the adult, and from three and a half to four inches in the boy. He also represents the passage of the urine from the kidney into the bladder to result from an active contractile movement of the former organ; and, that its expulsion through the urethra is accomplished by the muscular powers of the body of the bladder, overcoming those seated at the neck;—there being two instinctive and antagonistic powers concerned, corresponding in operation and result to those engaged in liberating the gravid uterus of its contents in parturition. The abdominal muscles assist the propulsive, and the perineal muscles the constrictive powers of the organ.

*General Pathological Observations.*

Diseases of the urinary organs often present symptoms referible to the urethra, while this part neither during life or after death manifests any corresponding change of structure. There are two classes of cases of this description: viz.

A. *Diseases of the Urethra, depending upon a pathological Condition of this Canal, without obvious or permanent Change of Structure, i. e. Spasmodic Stricture.*—The sudden and temporary nature of the stricture, and the absence of discharge from the urethra, point out this affection. The slow introduction of a bougie is easily accomplished, while, if this be passed roughly, the re-action of the canal may give rise to the belief of the existence of permanent stricture. Spasm often complicates permanent stricture, and is often induced by caustic, excessive distension, &c. &c. Too frequent coition, sudden application of cold, mental impressions, injuries to the perineum, the pressure of the child's head during labour, are causes. In the simplest cases baths, emollient and anodyne glysters, the immersion of the glans in cold water, or leeches to the perineum, are efficacious remedies: but when they do not prove so, and the suffering is great, or retention of urine menaced, we must have recourse to the bougie or to the catheter, a remedy when carefully employed neither difficult or dangerous.

B. *Affection of the Urethra resulting from the Influence of Sympathy with other parts of the Body: Neuralgia of the Urethra.*—The causes of these neuralgic affections of the urethra are often very obscure: occurring frequently in those who have become debilitated by venery, or who are suffering from some disease of neighbouring organs, they are found in others who are exempt from any such predisposing causes. The symptoms frequently resemble those of the most serious diseases of the urinary organs, and are chiefly discriminated by their sudden access, variability, and intermittence—sometimes observing exact paroxysmal periods. Although, when the neuralgia exists as a mere complication and aggravation of some other important disease, its relief may become a matter of difficulty, yet, when existing in its simple state, this is not usually the case. It is chiefly effected by the wax bougie; but, when this instrument is employed without address, it gives great suffering, and often leads to the erroneous belief that permanent stricture exists, and undue credit is awarded for treating the imaginary complaint. The author long since observed, that the exalted sensibility or irritability, frequently present in the urethra in calculous and other affections, became relieved by this instrument. He introduces a middle-sized bougie daily, and leaves it in the urethra from five to ten minutes, encreasing the size slowly, accordingly as the patient is enabled to permit this, and continuing the applications until all irritability has subsided. In obstinate cases, the introduction of a metallic catheter or lithontritic instrument has expedited the cure. Joined with this means we must pay due attention to the condition of the alimentary canal, correct the acridity of the urine. Cold douches to the perineum, hypogastrium, &c., the excitement of antimonial pustules, and, in very obstinate cases, a gentle application of the nitrate of silver, are other means. Some cases resist all means of treatment however varied. Neuralgia of the urethra is by no means rare in women, in whom however it presents very varied characters, and is usually confounded with affections of the neighbouring organs. Neuralgia of the urethra may complicate any disease whatever of the genito-urinary organs, and render its treatment inert or even impossible, until it has itself become relieved.

### PART I.—PERMANENT STRICTURE OF THE URETHRA AND ITS CONSEQUENCES.

### CHAP. I.—*Organic Lesions resulting from Stricture.*

A. *The Lesions which constitute the Stricture itself.*—These are found to occur in the form of *brides* or bridles, which do not however result as has been supposed from ulcers or cicatrices; and as *excrescences or vegetations*, so needlessly subdivided by authors, existing in every variety; but a *thickening or induration* of the walls of the urethra is the most common condition of parts to meet with. In some cases the morbid tissue becomes changed in colour, but in most cases there is no change in colour or density of the submucous tissue, the induration seeming to result from a mere increase of substance. Sometimes not only does the canal lose its elasticity but acquires a horny texture, and the dryness, occasioned by the absence of mucus, causes additional pain during the passage of instruments or urine.

B. *Lesions which are the Consequences of Stricture.*—(1.) *Phlegmasia of the Mucous Membrane behind the Stricture.* That this should occur from the irritation of the walls of the urethra distended by the urine, forcibly propelled by the continued contractions of the badder, will at once be seen. In truth this is the seat of the irritation, erroneously believed to exist in the actual stricture; for, as the narrowing of the canal encreases, the inflammatory action, which may have originally produced it, seems to become displaced, and is found at a point posterior to it. It is from this part the chief discharges proceed. Mere redness or depositions of lymph may be found after death, or there may be breach of surface, and the mucous membrane is sometimes perforated like a sieve. (2.) *Abscess of the Walls of the Urethra* will be noticed separately. (3.) *Dilatation of the Urethra behind the Stricture* may vary in extent, from being hardly perceptible, to a cavity large enough to hold an egg, and which has been mistaken for the bladder itself. All things being equal, the extent is proportionate to the hypertrophied state of the walls of the bladder, and to the degree of resistance offered by the stricture. It occurs independently of breach of surface, and, in the majority of cases, is confined to the membranous portion of the urethra. The prostatic portion becomes sometimes affected, involving part of the gland in atrophy, and the mucous follicles at the neck of the bladder, and the extremities of the ducts opening into the urethra, become dilated in like manner. (4.) *Rupture of Urethra behind the Stricture.*—The apertures vary in form, extent, and direction, and, occurring sometimes spontaneously, they are usually preceded by inflammation of the mucous membrane. (5.) *Formation of Sacs in the Urethra.*—These little cavities, varying in number, are found especially at the lower and lateral parts of the membranous portion of the urethra. They are usually covered by a fine membrane, and their existence is not incompatible with considerable duration of life. (6.) *Changes in the Urethra anterior to the Stricture.*—Although it is posterior to the stricture that the urethra usually becomes affected, yet this is by no means exclusively the case, for from sympathy or continuity of tissue it may become inflamed anteriorly. (7.) *Lesions of the Prostate.*—Although it is common enough to find this gland in a diseased state in stricture, it is by no means a necessary consequence, and may be a mere complication. The author has in numerous examinations, in very bad strictures, even occasioning retention of urine, found the prostate in a normal condition; while, in the aged, the gland frequently undergoes great change, unaccompanied by stricture. Still, doubtless the irritation of its mucous membrane caused by the detention of urine in stricture, often is followed by abscess in its substance. (8.) *Lesions of the Genital Organs.*—The production of hernia humoralis will be afterwards considered. The tumefaction of the *prepuce* is a common occurrence, when lesions exist at the prostatic portion of the canal, or at the neck of the bladder. In these cases, the penis often acquires a very unusual development, becoming elongated, hard, and rigid, a condition not to be confounded with the flaccid elongation resulting from masturbation, and traction of the penis by sufferers from stone. (9.) *Lesions of the Bladder* will be treated of at large hereafter, modifying as they do the symptoms of stricture in a remarkable degree. (10.) *Lesions of the Ureters.*—Civiale has frequently found these distended and inflamed, but not usually thickened. The distension may occur on one side, or only at intervals, giving the appearance of nodosities. Sometimes narrowing of the canal analogous to that of the urethra occurs at intervals. (11.) *Lesions of the Kidneys.*—The greater part of the patients who die, perish from consecutive disease of the kidney.

### CHAP. II.—*Seat of Stricture.*

When an acute state of inflammation of the mucous membrane passes into the chronic stage, some portion of the canal becomes modified in its vitality, and the submucous tissue encreased in quantity and consistence. The canal is not only diminished in size at this point, but loses its dilatability, elasticity, and suppleness. There are various parts of the canal especially liable to this occurrence, viz. the orifice of the urethra, the two extremities of the fossa navicularis, the anterior part of the spongy portion, and the junction of the bulbous and membranous portions—so that, it may be found sometimes at the commencement of the canal, sometimes at the depth of 3½ inches, and at others at the depth of 5. The nature of the stricture often varies according to the part in which it occurs: thus, at the orifice it is frequently a mere bridle, interfering little with the suppleness of the parts: at the spongy portion it is usually a broad, flat, resisting band, accompanied by induration and thickening; while, at the curve, it is frequently linear, as if from a ligature, and the neighbouring mucous membrane may be covered with vegetations or rugosities. No rule as to the *duration* of stricture can be laid down, for, although the cure is usually proportionately remote as the disease has existed long, some remarkable exceptions to this exist. Where there are several strictures, their number can frequently be only determined as we remove them, and not from the use of an instrument, or the manner in which the patient urines.* The most considerable is often found at the curve, and others less so between this and the glans.

### CHAP. III.—*Diagnosis.*

Easy as this may seem at first sight, errors frequently prevail, and we must endeavour to form a correct diagnosis by a consideration of the functional disorders, and the organic lesions, and by exploration.

A. *Functional Disorders.*—These are less the immediate consequences of the stricture than of the difficulty which supervenes in the expulsion of the urine—a difficulty that may be produced by other causes than stricture. (1.) *Discharges from the Urethra.*—The phlegmasia, giving rise to stricture, produces a puriform or mucous discharge, but, according to the stage, &c. of the phlegmasia, its appearance varies so much, that its source can oftentimes be scarcely judged of. The discharge sometimes becomes obstinate, owing to the too early discontinuance of the means of treating the stricture. It is not enough to restore the mere diameter of the canal; for, owing to the change in its vitality, the transition from the perverted

* We would suggest the employment of the verb "to urine" (for which we have the authority of Dr. S. Johnson), as avoiding much circumlocution.

to the normal condition, and the re-establishment of its suppleness and elasticity, require a long duration of treatment to effect. Then, again, when the prostatic and the seminal ducts participate in the inflammatory action, or, when sacs are developed in the urethra, the case becomes still more obstinate. (2.) *Incontinence and Retention of Urine* will be alluded to hereafter. (3.) *Hæmaturia* is observed in some old strictures, united with great distension of the walls of the canal. (4.) *Catarrh of the Bladder.*—This is a very common occurrence in stricture, arising from a chronic phlegmasia of the lining membrane of the bladder induced by the violent contractions of the organ, the acridity of its contents, or great distension. Sometimes it occurs when the stricture will permit a middling-sized bougie to pass, and thus causes attention to be taken off from the state of the urethra. (5.) *Impotence.*—Erections are rare, and the emission of the semen performed slowly, incompletely, painfully, and often only guttatim. After emission, however, the patient frequently urines more freely. The existence of the obstruction is not the only cause of impotence, for the inflammatory action often extends along the seminal ducts to the testes. The cure of the stricture sometimes restores the lost power of procreation. (6.) *The Organic Lesions* that can be discovered during life are few in number. The irregularities that are felt in the course of the urethra in perineo are not signs of stricture, but relate rather to the bulbous prolongation, or to the contracted state of the perineal muscles, for, the canal here lies too deeply to discover any such prominences by the finger. *Infiltration of urine* is a confirmatory proof, and it is an extraordinary fact that such infiltration may occur without any aperture in the urethra. The rigid condition of the walls of the canal seems sufficient sometimes to induce it, for it occurs when the stricture will admit the passage of a tolerably-sized instrument.

c. *Exploration.*—Catheterism is a difficult operation in stricture, and experienced surgeons have failed to reach the bladder, even when the urethra has continued in its normal state. Obstacles to the passage of the instrument have too frequently been set down as stricture. The disease is indeed often produced by the abrupt passing of instruments by inexperienced practitioners, and, when existing, has become aggravated by the same proceeding. Civiale introduces a wax bougie *very slowly*, by which means he has often succeeded in passing an instrument, that the urethra has rejected when hastily introduced. By the bougie an exact impression of the stricture, a temporary dilatation, and a diminution of the morbid sensibility of the canal are produced. If the wax coating be too hard, it will be pealed off in passing the stricture.

Chap. IV.—*Causes of Stricture.*

The greatest confusion has prevailed in the enumeration of these. Some, as tumours in the course of the canal, produce deviations rather than obstruction; other supposed causes, as obstinate gleet, are only consequences; and of others we have no proof, as a varicose state of the urethra, and neck of the bladder. The only circumstances we are justified in considering as causes, are those capable of producing and maintaining a state of irritation in the urethra. Among these we may mention (1.) *The Abuses*

*of Coitus* and prolonged erections: but, although these have undoubtedly some influence, this is much less powerful and general than is believed. (2.) *Gonorrhœa* is a very frequent cause, for, although it is an exaggeration to say, with some, that it is the only cause, yet, is the error on the other hand, of entirely denying its influence, as great. (3.) *The Use of Instruments.*—Next to gonorrhœa in frequency as a cause of stricture, may the means used for its relief be mentioned. Instruments, forcibly employed by the ignorant, will frequently be arrested in their progress through the canal, although no stricture may be present. (4.) *Violence* inflicted upon the urethra from within or without. Stricture, of an obstinate character, may rapidly follow the distension or laceration of the parietes of the urethra by a calculus, blow on the perineum, &c. (5.) The cicatrix left after lithotomy, especially when the convalescence has been tedious. (6.) A calculus detained in the membranous portion of the urethra may excite irritation, and develop a true stricture.

### CHAP. 5.—*Treatment of Stricture.*

This is general or local.

A. *General Treatment.*—The delays which patients frequently subject themselves to often render the affection much more complicated, and difficult of treatment. Thus there may be an encreased degree of local sensibility, an exalted condition of general erethism, or a deranged condition of important organs as those of digestion, &c. Most patients abstain from *drink*, hoping thereby to diminish the quantity of urine; but they also render it more acrid, and thus encrease spasmodic contraction. Mild drinks should be taken in sufficient quantity to render the urine limpid. The indulgence in *coitus*, when not excessive, does not seem to be attended by the ill effects mentioned by authors. *Bleeding* may be required, but it is usually most efficacious employed *locally*, as by leeches to the anus, hypogastrium, or perineum. *Baths*, both general and local, frequently repeated and prolonged for a long period, followed by emollient and sedative cataplasms, lotions, &c. are also useful. The local excess of sensibility is to be met with opiated glysters and suppositories, and the slow introduction of soft bougies. In this way many cases may be relieved.

B. *Local Treatment.* (a.) *Temporary Dilatation.*—This is accomplished by bougies. The author prefers the wax to the flexible metallic instrument. He disapproves of the conical form, for, the wider portion acts upon the part of the urethra that requires no dilatation, and the narrower frequently irritates the urethra, or gets involved in false passages, or the valves of the canal. It should be cylindrical, until within an inch of its termination, when the size ought gradually to diminish, and it should terminate in a smooth and rounded extremity. The urethra is less long than is generally supposed, and a bougie of ten inches, after its introduction, will have $1\frac{1}{2}$ inch beyond the orifice of the urethra, and one inch within the bladder. In introducing the instrument we must not bring it too precipitately down upon the stricture, for this will only irritate the urethra, and prevent our learning the exact seat of the affection; and this single error has frequently defeated all attempts at relief. If too great delay occur, however, the bougie becomes prematurely softened. The rotatory movement is hurtful, as it may irritate the urethra, and change the form of the bougie. Sometimes difficulties present themselves, not to be overcome by the ablest hand; but, usually, when obstacles exist, the letting the extremity of the instrument repose in contact with the stricture for a while, is attended with excellent effect. Civiale supposes this arises from the morbid sensibility or state of spasm, so commonly co-existing with stricture, becoming relieved; and examination after death has often shewn that a stricture might be traversed by a bougie, which had refused a far smaller one during life. This explains, too, the benefit of passing down to, and leaving for a while in contact with the stricture, a bougie of considerably larger dimensions than the one intended to traverse the stricture. The author blames the practice, however, of allowing hard sounds to remain in contact with the parts for hours or days, their pressure being sometimes even encreased by mechanical force. He is surprised that so few surgeons avail themselves of the valuable information, derived from the impression made by the stricture on the soft bougie.

The slight pain or uneasiness felt on the first introduction becomes diminished each time of repeating the operation, providing that the cure be not too much hastened, and that the size of the bougie be not too abruptly encreased. The instrument should be retained, at first, for a very few minutes only, but the time may be afterwards lengthened to half an hour. As the treatment cannot be considered complete until the normal diameter of the canal is re-established, in order to admit a proper sized bougie, the orifice of the urethra may require incision, for the attempt to accomplish it by means of a fusiform bougie, without previous incision, frequently gives rise to great irritation. Incautious use of the bougie may give rise to re-action and various inflammatory consequences, and before experience had taught the author the necessity of the greatest caution, he found retention of urine, urethritis, spasm, and febrile re-action frequent occurrences; but, since he has employed greater precautions, such ill consequences scarcely ever occur. Indeed, the objections which have been taken to the use of the bougie are chiefly applicable to its improper employment. This instrument when properly employed is advantageous from the facility with which it penetrates the stricture, the little pain and irritation it causes, and the model of the progress of the stricture it presents; the slowness and brief duration of its application and dilatation favouring the gradual restoration of the vitality of the part, the resolution of the engorgements, and the restoration of the suppleness to the urethra. By its use the violence, frequently producing false passage, retention, or ruptured urethra, when the sound is employed, is avoided.

(b.) *Permanent Dilatation.*—When the stricture will not admit the passage of a soft bougie, a small catheter must be introduced: Dr. Civiale prefers the gum elastic, having a shorter but more sudden curve than that generally employed possesses, the extremity not being pointed. He gives minute instructions for introducing this instrument for which we have not space. In the *normal condition* of the canal, he says, as the greater number of the lacunæ are situated upon the dorsal aspect of the canal, the instrument should be directed along its inferior surface: he blames the *tour-de-maitre*, as a piece of charlatanism; and a sudden rotatory movement practised at so narrow a portion of the urethra, especially if much pressure be employed, is very dangerous. We should proceed slowly, especially as we approach the pubic arch, where the sensibility is greater.

The manner in which the urethra may become distorted by a stricture, which occupies only a part of its diameter, and which cannot be accurately pre-known, shews us the folly of the advice which recommends the pushing on the catheter in the direction of the canal. Owing to the very yielding state of the parts supported by the sub-pubic ligament, the strictured part may sometimes be pushed forwards before the beak of the instrument; and, as the sensation given to the hand resembles that derived from passing this narrow portion of the canal, if the handle of the instrument be continually depressed under the delusion that its point has passed the stricture, a false passage in the superior wall of the urethra may be made, as has indeed often occurred. When complete *retention* is present, too much disposition to employ force frequently prevails. The author carries a small instrument down to the obstruction, against which it may remain applied for some instants, the object being to get it engaged within the stricture. If this cannot be accomplished a smaller catheter must be employed. When it has once entered the stricture a gentle pressure in the axis of the urethra, accompanied by traction of the penis, must be employed. When the resistance is very great, and contraction occurs at intervals, the exertion must be suspended, and again renewed; in this way cases are often relieved that resist more active manœuvres, one case cited occupying four hours in the introduction. No shocks, violence, or sudden movements are admissible, the canal should have time, to use the author's phrase, to *swallow* the instrument. The flowing of the urine is not always a proof that the catheter has reached the bladder, as this may occur even before it has passed the membranous portion.

Although great relief attends the first introduction, yet the leaving even a gum-elastic catheter in the urethra often excites great irritation, and sometimes excessive suffering, and general fever. Some degree of inflammation, which may extend to other organs, is almost always developed. The strictured part, however, becomes softer, and more extensible, and admits larger and larger instruments. It is erroneous to state that the catheter acts merely mechanically as a wedge, but more correct to suppose it modifies the utility and nutrition of the part, and thus removes the deposition. But by this means the walls of the urethra are maintained in an artificial state of dilatation, very different from their naturally approximative condition, and thus permanent is far less useful in res oring the natural elasticity of the parts than temporary dilatation, and relapses after its employment are more frequent. It requires too the confinement of the patient, and that for a long period, and risks the production of serious accidents; so that it should only be had recourse to when retention demands it, and not merely for the practitioner's convenience.

c. *Caustic.*—Great variety of opinion as to the employment of caustic has prevailed at different times; and thus, in England, where the practice sanctioned by Hunter, was so long enthusiastically followed, this means of treatment is now less followed than in any other country. It has been applied, too, with very different views; some seek to modify the vital properties of the urethra, and soften the obstacle so as to admit of future dilatation: others aim at the destruction of all morbid products, and blame future dilatation: some think caustic only adapted to linear strictures, and others reserve it for those that occupy a considerable portion of the parietes: some apply it very lightly, and others act deeply with it: some recommend it only when the situation and character of the stricture are distinctly ascertained, while others attack all strictures indiscriminately, believing such to be the quickest mode of forming a passage. The author states that a simple bridle may be removed by one caustic application, and an instrument passed which could not have passed before. In a more extensive stricture, whose exact nature has been learned by the bougie, two or three very slight applications of caustic often act beneficially. But, when the stricture is very considerable, a first application often produces very slight effect, while, if frequently repeated, it may give rise to numerous evils. Where there have been several strictures, the application of caustic to the anterior one has sometimes relieved those posterior to it, with which this substance never came into contact. The relief derived is rarely permanent. Caustic has been erroneously supposed to act as an escharotic. If it did so the mucous membrane would be seriously injured, but the disease does not exist in this membrane, but in a change of the tissue situated beneath it. After the application, a slight redness and swelling surrounds the greyish spot of application; in a few days a thin layer falls off, a greater activity of capillary circulation follows, and a softening of the strictured part in consequence of the modification of its vitality takes place. In a very narrow stricture it becomes impossible to apply the caustic to the part where it is required, and, when incautiously used, false passage, infiltration, affection of the generative organs, &c. may ensue. When the substance has been frequently applied great difficulty of passing water results, not from the re-production of stricture, but from the rigidity of the walls of the urethra, this induration being sometimes so great as to resist powerfully the passage of the catheter. At other times irregularities, cicatrices, and excessive irritability of the urethra exist.

D. As Dr. Civiale, by patience and time, has always hitherto succeeded in penetrating into the bladder, he has never had recourse to various other means, such as dilatation by injection of air or water, scarification, incision, forcing, &c.: but, judging from the reports of others, he is not disposed to think well of them. He has never found it necessary to puncture the bladder.

Chap. 6.—*Varieties of Stricture.*

The author prefaces the consideration of this subject by some remarks upon certain pathological conditions of the *prepuce, glans* and *penis*, which sometimes complicate affections of the urethra. The *prepuce* in some persons nearly covers the glans: when this latter can be sufficiently exposed to enable a full-sized catheter to be passed, surgical interference is usually unnecessary; but, when a mere probe can be passed, the sebaceous matter which accumulates beneath the prepuce, sometimes exerts a powerfully compressing effect upon the urethra, preventing the due expulsion of the urine. The necessary incision should not be performed at the dorsum, as this gives rise to a deformity, frequently painful during coition. Phymosis is usually congenital, but it is sometimes brought on by irritation of the glans or urethra. The ulcers occurring beneath the prepuce are usually due to a want of cleanliness. If neglected, the prepuce may become thickened and indurated, and the glans atrophied, while the ulcers sometimes take on a cancerous action. Dr. Civiale, in affections of the prostate, or of the neck of the bladder, has frequently found the glans and corpus cavernosum of the penis so swollen and indurated, as to give rise to the appearance of the presence of an inorganic body. This sometimes presents a serious obstacle to the passage of instruments. This state must not be confounded with mere want of elasticity in the urethral walls, although the two conditions may become combined.

A. *Bridles at the Orifice and anterior to the Fossa Navicularis.*—A small bridle or two existing at the orifice may be easily divided. The narrowing of the urethra before the fossa navicularis, is sometimes increased by the development of a membranous bridle at its lower part, which, as it does not cause much difficulty to the expulsion of the urine, may have acquired considerable size and thickness before it is discovered. Its existence encreases the liability to the formation of calculi in the fossa, and renders the treatment of other strictures lower down the canal difficult and painful. The treatment of this by dilatation or caustic is unsatisfactory, and the author divides the bridle with his *urétrotome* and introduces a large bougie for some seconds every few hours. It is better to repeat the incision once or twice rather than make too deep a one at once. It is surprising to observe what serious symptoms, and how altered a state of the canal will be relieved by this slight operation, proving that an obstacle to the expulsion of urine, which is hardly perceptible, may yet give rise to the most serious local and general consequences. The cure is slow and gradual, but more certain than by any other means. Sometimes the stricture is seated just posterior to the fossa.

B. *Stricture in the Spongy Portion of the Urethra.*—It is here we usually find examples of extensive stricture, and which is usually not susceptible of perfect cure. Violence done to the urethra by dilatation, removal of calculi, &c. is a frequent cause, and little time is often required to develop such a stricture. Sometimes it rapidly follows gonorrhœa, or the use of caustic. The author recognises two degrees of the affection. In the first the wax bougie is to be employed, not retained permanently in the urethra, but only from a few minutes to half an hour, or a few slight applications of caustic may be used. In the second, where the stricture has become dense and callous, and frequently incurable, the caustic is only hurtful, and the temporary dilatation often inefficient; and one of the characters of this affection is the forcible manner in which it embraces the instrument, and thus renders its removal and re-introduction so difficult. This anormal contractility has, however, occasionally given way to the persevering use of bougies, commencing with a very small one. It is in these cases by permanent or prolonged dilatation, that most benefit is derived. Division or scarification of these strictures, from a supposed analogy existing between them and the bridles of the orifice, has been employed by some: but even if successful as regards the stricture, this means and the cautery leave the urethra in such a state of rigidity, that although a good-sized instrument may be passed, yet dysury, strangury, and paralysis of the bladder may result, as also various lesions of the prostate, vesiculæ seminales and testes. Old strictures in this part of the canal possess a density, resistance, contractility, and a tendency to relapse, not remarked in the deeper portions of the canal: and, thus, although the facility of the application of means is greater, the cures are less easy, certain, ready, and durable.

c. *Stricture at the Curved Portion of the Urethra.*—This, by reason of the natural narrowing of the urethra that here takes place, and the injury done by the inexpert passing of instruments, is the most frequent site of stricture. It may be described as occurring in two degrees: the slighter, in the form of a mere linear stricture, like a semicircular fold of the mucous membrane, opposite the subpubic ligament, is often attended with little inconvenience, save perhaps an encreased frequency of desire to urine, and the presence of an obstinate gleet. Caustic has acquired much of its celebrity by the cure of these simple cases; but the author thinks the case is far better treated by temporary dilatation, with the wax bougie, and, on no account would permit of permanent dilatation. But the patient frequently does not apply until a dense mass of substance surrounds the stricture, through which the merest aperture exists, and accompanied by the severest symptoms, even complete retention. Here the mere exploration of the mischief with a small bougie is exceedingly difficult, requiring great manual dexterity. When the stricture is found to be very long and hard, it will be best treated by an elastic gum catheter. The change of the size of the instruments must be most cautiously proceeded with, for the stricture holds them with a great retaining power, and the re-action and irritability produced by their withdrawal may become excessive. Where there is vesical catarrh, hæmorrhage from the bladder, retention, &c. the case must be treated by the permanent dilatation of the catheter, but, as soon as such complications are subdued, the bougie should be resorted to. So, too, a succession of hard, callous structures, false passages, deviations from the normal direction of the canal, &c. may require the substitution of a catheter. The case becomes much complicated when a calculus exists behind the stricture, for, in these cases the urethra is usually very irritable, and the patient objects to the use of instruments, on account of the pain they inflict. The stricture prevents the escape of the calculus, while the calculus irritates the stricture. If the bougie here proved unavailable in such cases, the stricture must be forced by a catheter, and the calculus thrust into the bladder, or an incision must be made in perineo, according to the state of the stricture and of the surrounding parts, of which an examination by the rectum will assist in forming the judgment. In these cases, too, the author has employed dilating forceps with success, but protests against such examples justifying the application of the system of forcible dilatation to the treatment of stricture in general. We may have to substitute the catheter for the bougie in some other cases besides those mentioned, as in obstinate induration of the urethral parietes. The urethral valves, too, by entangling the extremities of the bougie, sometimes give rise to the erroneous belief of the existence of stricture. Some patients, when introducing a bougie for themselves, bear too much upon the inferior wall of the urethra at the point of union between the membranous and prostatic portions, so that a kind of excavation may become formed between the prostate and rectum, giving rise to the belief of an additional stricture. An examination by the rectum detects the vicious course of the instrument, which may be rectified by giving the bougie a sharper curve, or substituting an elastic catheter with its stilette. If a passage to the bladder cannot by any means be obtained by instruments, a case never yet met by the author, he advises the simple division of the membranous portion of the urethra, to liberate the urine, but condemns, in the altered condition of the stricture and surrounding parts, the attempting the division of the strictured part—a proceeding both difficult and dangerous. In cases, even, where with skill, time, and caution we have arrived at the bladder, and our end seems accomplished, the patient may sink under the shock occasioned by what has been done for him.

CHAP. 7.—*Relapse of Stricture.*

Although we may have succeeded in relieving a stricture, yet, this being only an effect of a diseased state, we must to prevent a recurrence remove the cause; the very means, indeed, we employ in relieving the stricture may produce such a condition of the canal as to lead to its reproduction. It is too customary to consider the narrowed urethra as a mere inert canal, for the enlargement of which mechanical force is alone requisite, taking no account of the loss of elasticity in its walls which constitutes so important an item in the affection, and which permanent dilatation is so likely to aggravate. Thus relapse is far more frequent after treatment by this means or by the use of caustic, than it is when temporary dilatation by the wax bougie has been employed; and, when it is found at all after this latter, it generally arises from the treatment having been too rough and hurried, and not continued over a sufficient space of time. The tendency to relapse is greater in proportion to the extent and density of the stricture, as the cure is in these cases often very incomplete. A relapse after the use of caustic produces sometimes a more serious disease than the original one.

CHAP. 8.—*Affections resulting from the Presence or Treatment of Stricture.*

1. *Discharges from the Urethra.*—When the urethra has once undergone, from prolonged inflammatory action, even a slight organic change, a very slight cause will renew the phlegmasia with attendant discharge. Errors of diet, a ride on horseback, or coition will suffice; and thus many men who lead irregular lives pronounce every woman they meet with as infected, while many a family quarrel has arisen from the same cause, and has been fomented by the want of discrimination of the medical adviser. Sometimes discharges are produced by the connexion of two entirely healthy persons, independently of any excess, malformation, or want of proportion. Discharges arising from stricture, contracted orifice, phlegmasia, accidental lesions, &c. take on every variety of character, and are often accompanied by very anomalous symptoms. In treating these cases we should attack the cause producing it rather than the discharge itself, and the neglecting this rule, has given rise to so much empirical treatment, often serving only to encrease the original malady. The discharge in stricture was formerly deemed beneficial, as indicating a solution of the obstacle, and it is certain that such discharge is usually proportionate to the degree of dilatation produced; and in those callous strictures, in which no discharge appears, no progress towards cure is made. When, however, it becomes excessive, we must proceed more slowly, and adapt our instruments more carefully. So, too, the entire cessation of the discharge after the last bougie is a proof of cure.

2. *False Passage.*—When we consider the form, extent, situation, and density of strictures, the frequently morbid condition of the canal anterior to them, the form and size of the instruments intended to traverse it, the mode of action of some of the special means employed, and the small number of surgeons sufficiently exercised in catheterism:—the numbers of false passage met with in practice are far less than might have been expected. Their nature and extent will vary according to the means by which they have been produced, and the structure of the parts they have implicated. In the spongy portion of the urethra they are frequently produced by the patient endeavouring to treat himself, or by the practitioner employing a too-pointed instrument, and they may proceed to a considerable extent without detection. But it is at the curved portion of the urethra that we find false passage most frequent, the inferior wall being that which generally is perforated. The false passages produced by caustic are more extensive in surface but less deep than those produced by instruments, especially when these are conical. It is not always easy to detect when a sound penetrates the urethral walls, for the affected part often offers more resistance than the healthy portion—a lesson to those who employ force guided only by the resistance they meet with. The sensations of the patient furnish no guide, for the great sensibility of stricture is quite imaginary. The presence of hæmorrhage, and the sense of tearing, when the prostate is concerned, are very inexact signs. The change of direction of the catheter would advertize the surgeon that its beak had entered a false passage if this lay in an opposite direction to the urethra; but the passage is usually nearly parallel, and may be only just separated from it, and even the examination by the rectum will not enable a person, unaccustomed to such cases, to detect a deviation from the natural route. It often happens, on the one hand, that a catheter, although maintained in a straight direction, reaches the bladder only by perforating the prostate, and, on the other hand, it may sometimes become buried in the urethra to its very hilt without a drop of urine flowing. A surgeon is placed in great difficulties, when called to a case, in which the false passage has already been produced by the attempts of those who have seen it before him, for he incurs the risk of being supposed the author of it. However experienced he may be, he will often find it very difficult to decide whether the instrument has entered the false route, or whether it continues within the natural passage. Sometimes, however, even when a false passage exists, the instrument at once passes by the natural one.

The consequences of false passages are serious according to their situation, extent, direction, the importance of the tissues they traverse, the nature of the means by which they have been produced, and the state of health of the individual. If, as soon as the change of direction of the instrument is perceived, its further progress be at once checked, immediate serious consequences need not result. The patient may not be aware of the accident, and the chief ills resulting from it are the encreased difficulty of catheterism, and the delay, or perhaps the entire prevention, of the cure of the original affection. Such cases are continually occurring. When the walls of the urethra are pierced, and some important organ transfixed, the most serious or fatal results may follow in consequence of the infiltration of urine, and the excitement of inflammation. But, even here, a gum-elastic catheter, left in the false route, by removing the urine, and preventing its exciting irritation, gives time to the organizing of a new canal, and, even when important organs have become implicated, serious results do not always follow. In other cases, too, where death does not occur, a fistulous communication is maintained with the organ transfixed, *e. g.* the rectum. But, under other less favourable circumstances, a mere puncture of the walls of the urethra, extending to a very few lines in depth, may, if the egress of the urine from the urethra be obstructed, give rise to fatal infiltration.

3. *Infiltration of Urine.*—Under many circumstances the urine is brought into contact with tissues not intended for its reception: but the effects which result, and the manner in which the urine is to be diverted from its anormal course, vary in different cases. In some, the patient having a stricture, suffers all the symptoms of retention of urine, and a tumor has manifested itself for some days in perineo. This suddenly bursts, and all its contents are effused into surrounding tissues. It is an urinary abscess which has broken, and the effusion of urine is accompanied by a sense of pain and burning. In another case, where no external tumor appears, the patient may have a sense of something giving way, and of the escape of urine, although no sign of this be externally visible. Immediately all the dreadful consequences of the infiltration of the cellular tissue by this fluid supervene, and that in so sudden a manner, that the surgeon on his arrival usually finds they have reached their most alarming height. The urethra, damaged by the inflammatory action, has given way behind the stricture.

The *diagnosis* is often difficult, for, in several cases of stricture, the passage of instruments causes a tumor in perineo, which may be mistaken for an urinary abscess, but which disappears in a few days. In general the aperture in the urethra at the commencement of the effusion is very minute. The disposition of the perineal fasciæ determines, in some measure, the course of the effused fluid, but yet the great difference which may be observed in these effusions, accordingly as they have occurred suddenly or gradually, leads to the belief that the influence of the fasciæ has been exaggerated. The effusion makes progress in proportion to the looseness of the cellular tissue of the parts, so destructive is the agency which the presence of the urine exerts upon this texture. However various means for the treatment of the affection may be vaunted, we must ever remember that the true difficulty consists in the complication of the original lesion with the effusion, and that the former may form a more difficult matter for relief than the latter. If the effusion be circumscribed, an incision must be immediately made through its length and depth, and when this is freely and effectually done, the entire relief the patient obtains, at all events for the time, is remarkable. It is not always easy, however, to indicate the exact point whereat the incision should be practised, as the principal swelling is sometimes situated at some distance from the seat of rupture, and if practised there the mischief would not be arrested. Two rules must be observed, viz. to act speedily, and not to fall into the error which has often misled young practitioners, of making the incisions of insufficient length and depth, owing to the illusion produced by the swollen state of the parts, an error the author himself fell into at the commencement of his practice. Incisions which seem of frightful extent, after the evacuation of the effused fluid, have the appearance of mere scratches, and, in proportion as we freely divide the cellular bands, the parts in contact with the urine become liberated from it. Frequently, however judiciously we proceed, the patient sinks, for certain organic lesions, heretofore caused by the stricture, and not immediately incompatible with life, become, during the shock which has now agitated the system, excited into a state of fatal activity. In some cases, especially when the operation has been delayed, the constitution does not possess vigour enough to enable it to throw off the slough, or to perform the necessary reparation; but, when such vigour does exist, it is astonishing, as Dessault long ago observed, to what extent reparation will take place. Chopart relates a case in which the patient was saved, although a portion of the urethra, the prostate and the tunica vaginalis were exposed.

4. *Urinary Abscess with perforation of the Urethra.*—The dreadful cases of infiltration already mentioned are happily rare, but we often meet with others, in which the process is slower and more circumscribed. One or more hard tumors are produced by the inflammatory action, occurring in the parts with which the urine is in contact. These tumors usually occurring in perineo, or at the root of the penis, may be found in the region of the sacrum, pubes, abdominal parietes, or even the sternum. The abscess sometimes becomes developed without obviously active cause, and, at others, in consequence of false passage or the use of instruments or caustic. The recognition is usually easy, but occasionally, owing to its deep situation, stationary condition, and the coincident trivial state of the stricture, much difficulty may sometimes occur, and thus it may be mistaken for abscess of another description, or for hernia, while its distinction from the spermatic abscess, occurring in the vicinity of the prostate, is yet more difficult. The progress of the abscess is very slow, indeed almost stationary, and its internal surface becomes sometimes, as it were, organized. But, sooner or later, it will be followed, at its bursting, with all the alarming symptoms attendant upon complete infiltration of urine, and should, therefore, as soon as its diagnosis can be assured, be opened, without waiting for fluctuation. The existence of these abscesses is not always easily explained, occurring, as they sometimes do, without any irritation having been excited in the canal, and when the stricture offered scarcely an obstacle to the expulsion of urine. The lesion is constantly situated at the membranous portion, varying in size and extent, and frequently seems to originate in inflammatory action, extending from the little sacs, which are often developed in the urethral parietes. Although slowly, the urine in these infiltrations frequently makes extraordinary progress, when it is considered that the natural canal is open, and that fistulæ are often also present. Our object should be to prevent the formation of fistulæ, which can only be done by incisions of an adequate freedom, to open a free passage for the urine.

5. *Urinary Abscess without apparent Perforation.*—These are cases in which an abscess, having all the characters of an urinary one from its locality and appearance, is yet found unaccompanied by any traces of a communication with the urethra. A certain degree of irritation, occurring at this deep portion of the canal, even when there is neither stricture or retention, may produce this. The accident is not rare, when a catheter is retained in the bladder, even without any intentions of dilatation, as in paralysis of the bladder, or after the high operation of lithotomy. Even the incautious use of a wax bougie in stricture has given rise to the same calamity, from the size being too suddenly increased, &c. Dr. Civiale has frequently found little abscesses existing between the prostate and rectum, evidently of old date, from their being lined by a mucous membrane. In the abscesses we are now considering, the most minute investigations have failed to detect the existence of an aperture in the urethra, and their occurrence must, therefore, be explained either by the transudation of urine through its walls, or by the propagation of irritation by sympathy or contiguity. Usually in these cases the urethra is very irritable, and bougies fail in rendering it less so, but this is not invariably so. The diagnosis is pre-eminently difficult, and they are often not detected even until after death. Sometimes the tumor is sufficiently appreciable, and sometimes after long lingering it takes on a sudden development. Although the swelling usually seems behind the stricture, the author has met with two cases in which it was anterior to it. When the abscess is superficial and circumscribed it is not dangerous, and frequently it is connected with small fistulous apertures in the scrotum, whence a fluid, resembling urine, exudes. Related to these abscesses are the collections of matter which form in the vicinity of the vagina, and between it and the rectum, and frequently lead to urinary fistulæ in women. These may arise from external violence, from any permanent irritation situated in the vagina, and occasionally from the act of coition, either by reason of disproportion of parts, or of the excessive sensibility of the organs. Several young women have been admitted into the Hôtel Dieu, from this cause, and in them the vagina has been found narrow and highly irritable.

6. *Abscesses occurring in various parts of the Body during the Treatment of Stricture.*—During the treatment of disease of the urinary organs, and particularly those of the urethra, pains frequently attack the limbs, and especially the principal articulations. These are frequently mistaken as arising from rheumatism or phlegmonous erysipelas. Inflammation occupying at first a broad surface, quickly confines itself to a particular spot, and rapidly gives rise to the generation of copious fetid suppuration. These abscesses should be early and effectually opened.

7. *Urinary Fistulæ.*—The abscesses which occur without communication with the interior of the urethra, although their discharge is sometimes so abundant, are not usually accompanied by fistulous openings; and even when the walls of the urethra are injured by instruments, or other means, fistulæ by no means necessarily occur. The abscesses resulting from false passage rarely become fistulous, and those which follow the use of caustic are cured as rapidly as the common abscess, especially if they have been promptly opened. But in most cases of urinary abscess, the escaping of the urine from an aperture formed in the urethra, prevents the formation of a cicatrix. Fistulæ vary in almost every particular as of situation, extent, number, tissues traversed, and quantity of urine passing. They may be complete, establishing a communication between the urethra and integument: incomplete or blind, when their course has no exit or penetrates into other cavities, which is rare. It is an uncommon thing for the fistula to have more than one internal opening, but the author has seen examples where the urethra has seemed perforated like a seive, all the apertures afterwards uniting to form a single passage. Numerous external openings, following a succession of small abscesses, are common, and Civiale has counted no less than 52 branches from the original fistula. These communications may sometimes extend to great distances, as to the hypogastrium, the thighs, or even the legs; but this state of things is only found in old, neglected cases. Commonly there is however but a single passage, which acquires a special organization, being lined by a new mucous membrane, while its walls form a ligamentous-like cord, to be felt externally, providing the induration of the surrounding parts be not too considerable. The change in these is not confined to any one texture, for, although the cellular tissue suffers in the first instance, the very bones themselves may become affected, and the author alludes to a case of necrosis of the pubis arising from contact with urine. The cellular texture becomes changed not only in density, but in volume, while it entirely loses its sensibility, so that it may be incised without occasioning any pain. When the urine escapes by a single aperture, there is seldom much induration around the fistula, even when it has existed many years, so that its own cord-like form may be felt; but when the fistulous tract branches out into many directions, great changes, in the cellular tissue especially, occur, producing great tumefaction and much irritation. Occasionally, the swelling and induration which have encreased slowly, remain for a long time stationary, and the patient may live for a long while, suffering only some inconvenience in progression. If, during this period, we can operate favorably upon the diseased condition of the urinary tracks, the tissues, though slowly, may regain their natural condition.

The treatment has occupied much attention, but is still very defective, and numerous cases remain incurable. The original cause producing the tumor has been too frequently forgotten; and the operation of excising the fistulous passages would be often rendered needless if attention were more directed to relieving or curing the primary affection. When an operation has been resolved upon we must never forget the deceptive appearance caused by the tumefaction of the parts, and thus extend our incisions to a too trifling depth.

Urinary fistulæ are not rare in women. Cancerous or venereal ulcers, calculi, pins introduced into the bladder, wounds or punctures of the vagina, contusions of the urethra, &c. have led to them. The last cause, produced by the pressure of the child's head during labour, is the most common of all. In general the effect of such compression is only temporary, but, when it has been severe or long continued, a portion of the urethra may slough away, and a communication between it and the vagina become established; such cases are erroneously called vesico-vaginal fistulæ, for, with few exceptions, the opening is not between the bladder and vagina, but between the urethra and vagina—a fact of importance in the curative point of view. A remarkable effect of the contact of urine with the other tissues is the contraction of the vagina which it causes, so as to leave in some cases the merest aperture. Narrowing of the vagina has been but little noticed by authors, although it frequently results from syphilitic inflammation, violence, abuse of coition, &c. A few months or even a few weeks are sufficient to develop it.

8. *Affections of the Spermatic Cord and Testis.*—Daily experience proves the extensive sympathic influence of lesions of the urethra and neck of the bladder upon the generative organs. An inflamed testis may be produced by any urethral irritation, such as an impure coitus, stricture, or the action of some of the means intended for the relief of this latter.

In most cases of swelled testis there will be found to have been some preceding or coincident irritation of the orifices of the prostatic and ejaculatory ducts, whether arising from the use of instruments and injections, severe stricture, or the special diseases of the prostate. Under these circumstances emission is accompanied by great pain and is followed by great prostration of strength, which may continue for several days. Men, also, having stricture, or diseased prostate, are liable to pollutions, and these occurring without any provocative and often without even erection.

Chronic inflammatory action of the testis has not occupied a sufficient degree of attention, arising as it frequently does, from the slighter urethral affections. Affections of the prostate, and neck of the bladder, often lead to atrophy, or to a stationary condition of engorgements. The application of caustic, and especially of scarification, to the urethra exerts upon the secretory and excretory organs of the semen a notable effect. The author had long observed in such patients the loss of power of erection, accompanied by pains in the testis, groins, &c.; and noticed afterwards that these effects were proportioned in extent to the severity and frequency of the application of these causes of irritation. The testis sometimes, in these cases, becomes excessively and obstinately irritable. In all cases which have become relieved, this has seemed to depend upon the improved condition of the urethra induced by the use of temporary dilatation, taking care that it shall be pursued very slowly and cautiously.

We must defer the remainder of our analysis till next number.

